# Multidimensional scale of perceived social support: evidence of validity and reliability in a Chilean adaptation for older adults

**DOI:** 10.1186/s12877-021-02404-6

**Published:** 2021-08-11

**Authors:** Cristhian Pérez-Villalobos, Juan Carlos Briede-Westermeyer, Mary Jane Schilling-Norman, Sergio Contreras-Espinoza

**Affiliations:** 1 Department of Medical Education, Universidad de Concepción, Víctor Lamas 1290, Concepción, Chile; 2 Department of Engineering Design, Universidad Técnica Federico Santa María, Avda. España, 1680 Valparaíso, Chile; 3 Statistics Department, Universidad del Bío-Bío, Avda. Collao, Concepción, 1202 Chile

**Keywords:** Social support, Scales, Psychometrics, Validity, Reliability, Factor analysis

## Abstract

**Background:**

Given the relevance of social support on the mental health of older adults, having an instrument to evaluate this variable is essential for research in the area. However, mainly, having instruments with suitable evidence of their psychometric properties is critical. For this reason, this study sought to evaluate the factorial and reliability structure of the Multidimensional Scale of Perceived Social Support within autonomous older adults from the Province of Concepción, Chile.

**Methods:**

We surveyed 399 older adults using quote sampling. They answered a Spanish version of the Multidimensional Scale of Perceived Social Support, plus a sociodemographic questionnaire. We performed confirmatory factor analysis using Weighted Least Squares Means and Variances adjusted estimation (WLSMV) to compare the factor models proposes by previous studies. To evaluate reliability, we calculated Cronbach’s alpha and McDonald’s omega.

**Results:**

The Confirmatory factor analysis found that the 3-factors models showed the best fist index between the models with CFI = 0.991, TLI = 0.989, and SRMR = 0.035, even though RMSEA were over the cutoff point. The factors presented reliabilities from α = 0.858 to α = 0.941, and from ω = 0.937 to ω = 0.972.

**Conclusions:**

The results support the existence of three factors for the Multidimensional Scale of Perceived Social Support (MSPSS), differentiating the support perceived from Family, Friends, and significant others. All factors present good or excellent reliability. This solution is theoretically consistent and coherent with the literature, and it presents evidence in favor of the use of MSPSS as a measurement to distinguish the support perceived source.

**Supplementary Information:**

The online version contains supplementary material available at 10.1186/s12877-021-02404-6.

## Background

### The challenges for older adults

In Chile, the last census found that older adults made up 16% of the population [1]. As in the rest of the world, it implies an achievement for the health systems, demonstrating an increase in life expectancy [2]. However, it also is associated with physical and cognitive changes that push older adults to change their lifestyle, roles, and social responsibilities.

Previous research in Chile about quality of life among older adults showed that social relationships are an important aspect in this stage of life and are positively valued. Although 35% of those surveyed stated feel socially isolated, which reflects the need to continue working on this aspect [3, 4].

Regarding physical illnesses, research shows it can significantly affect the presence of anxiety and depression in older adults [5–7]. Likewise, chronic illnesses tend to go hand in hand with disability and functional difficulties, which reduces activity and increases social isolation, which is a risk for older adults [8].

Certain stressful factors can cause discomfort if the person does not have coping strategies to suitably react to these. In particular, social support is an important coping strategy for older adults, among others [5].

### Social support

Social support can be defined as the availability of people on whom one can rely [9] or the amount of assistance received through interactions with other [8]. It relates with the belief that the person is cared for and considered, valued and loved by others, and is part of a communication network [9, 10]. Social support is a multidimensional phenomenon since it depends on the socio-political context of the person, their socialization process, and personal values, among other factors [8].

Social support is affected by its structure, direction, type and sources of support. The *structure* considers the size of the support network [11], whilst *direction* is related to the social support received and the social support perceived. The first considers the assistance provided to the person by their support networks, while the second is related to the individual’s perception that receives the support regarding the satisfaction of their needs [10].

The *type of support* refers to whether this is emotional (physical affection and care), informational (providing information and support), instrumental (concrete assistance, services, or material goods), or valuing (feedback for self-assessment) [8, 11]. Regarding *support sources*, those who provide or receive this support are considered. Here, family members, partners, friends, colleagues, neighbors and pets stand out [12]. Research has shown that quality of support in relation to its type has higher impact on perceived social support and wellbeing in comparison to the structure of support networks in older adults. Similarly, in Chile elderlies have access to other support sources which include religious groups and community support from neighborhood and municipal centers [13]. Having a heterogenous support network has also been linked to better quality of life among older adults [14].

Social support for older adults is a protective factor for stressful life events. It improves mental health and mitigates the effects of depression, anxiety, low self-effectiveness, stress, and social isolation [7, 8, 13, 14]. It is also associated to increased longevity among older adults [15], better quality of life in regard to health and healthcare behavior [10, 11, 16].

Ultimately, the presence of a support network for older adults will facilitate the development of protective factors surrounding physical and mental health, which will improve their quality of life and allow them to enjoy this period of life. This study focused on perceived social support, that is referred to how older adults assess their access to instrumental and/or expressive provisions [17], and it also aims to differentiate between sources of support for older adults that tend, in general, to be lost and reduced with age [6], which would allow generating interventions focused on maintaining active social relations in this stage of life.

### Measuring social support

For a while now, research has been interested in measuring social support in the population, given its importance as a protective factor for life. This contributed to the creation and validation of instruments that measured this construct. Some well-known instruments to measure social support are ISEL-12 (Interpersonal Support Evaluation List), the Social Support Questionnaire, Social Provisions Scale, and the Multidimension Scale of Perceived Social Support (MSPSS) [6].

The MSPSS scale is one of the most commonly used scales to measure this construct [8]. It was originally created and piloted in American university students to differentiate three sources of perceived social support: Family (FA), Friends (FR), and Significant others (SO) [17].

It is a quick application scale that is also easy to administer and use. This is a plus when the interviewee has a limited time for the application, especially when more than one scale is applied. On being short, it also avoids that certain answers are marked randomly due to tiredness and/or boredom [18–20].

### Validation of the MSPSS

Besides the MSPSS’s administration advantages, it is important to gather evidence about its validity and reliability in different populations since social support tends to vary depending on the age group. Likewise, cultural differences impact on the perception of social support, which could affect the structural validity of the instrument [8].

Several studies had tested its factor structure in different populations like university students [17], medical students [19], adult population [21], pregnant women [22], women in the postpartum period [23], cancer patients [24], teenagers [25, 26], patients with chronic illnesses [27–29], incarcerated adults [20], family caregivers [30], and older adults [3, 31–33], demonstrating excellent psychometric characteristics [3, 8, 18, 31]. The scale has been translated to different languages and adapted to socio-economic settings in different countries [8]. Also, it is one of the few scales that identifies the support source [6].

Most of the psychometric research has supported its 3-factors structure [17, 18, 20, 21, 23–27, 29–31], but some studies have found support for a 2-factors solution where Significant other’s factor merges with Family [3, 32, 33] or Friends [34]. Even a study has found a one-factor solution for the Hurdu version [22].

In older adults, Stanley found a 2-factors structure in a population with a generalized anxiety disorder (GAD) and a 3-factors structure for the control sample. In the GAD sample, the Significant others factor merged with the Family factor [32]. In Chile, Arechabala found the same structure using an exploratory factor analysis followed by confirmatory factor analysis [3]. Nevertheless, two later studies in this country, one in a sample of older adults and another in a sample in diabetic population with an average age of 65, showed support for the 3-factor solution [29, 31].

These discrepancies in factor structure may be affected by the individualistic or collectivist characteristics of each social setting studied, which allows identifying the diverse groups in a differentiated manner, or, on the other hand, grouping them into a single collective [8]. Also, it may result of age or health, where younger people tend to relate significant others to friends, and older people or people with physical or psychological dependence tend to relate significant others to family [19, 32, 33].

To face this problem, Wongpakaran and team added the note “Significant other excludes friends and family” in the MSPSS’s Thai translation in order to help respondents to distinguish significant others from the other groups. This modified version has showed good fit indexes for a 3-factors model in medical students [19] and older adults [33].

For this study, we used the Chilean translation of the instrument made by Arechabala & Miranda [3]. This version modifies answer options. In 1988, Zimet changed the 5-point Likert scale of this original version for a 7-point scale (1 = very strongly disagree to 7 = very strongly disagree) to increase responser variability and minimize a ceiling effect (Zimet), but in published studies, we can find both 5-point [9, 19, 21] of 7-point scale versions [23, 24, 26, 32–35]. All of them from a stronger disagreement to a stronger agreement.

But, in the pilot application of the Chilean translation, Arechabala found that older adults showed difficulties in using this agreement answers options. So, they adapted MSPSS to a frequency scale with fewer alternatives. This adaptation has been used in the three previous studies in Chile that have sought to validate this instrument in Chilean older adults [3, 29, 31]. These studies have certain aspects in common that represents limitations. All of them use exploratory factor analysis (EFA), which must be complemented or replaced by confirmatory factor analysis (CFA) [6], which only Arechabala’s research performed. They used samples below the suggested standards [36] and focus on the clinical population. The samples were obtained from primary healthcare centers and in the studies by the teams of Ortiz [29], and Arechabala [3] they included specifically diabetic or hypertensive patients, thus focusing on a very specific elderly population that have chronic illnesses.

All of this makes it necessary to perform a psychometric analysis of MSPSS in a Chilean older adults’ general sample, beyond the clinical population, using CFA.

In order to have complementary evidence of its validity and to assess how it behaves in heterogenous elderly population that reflects the reality of the country, as well as using larger samples, this study was set out to evaluate the factor structure and reliability of MSPSS in the autonomous older adults of the Chilean Bío Bío Region.

## Methods

A quantitative, psychometric study was run using surveys.

### Participants

The population comprised autonomous older adults from the Chilean Bío Bío Region. Inclusion criteria were defined so that these people were over 60 and were classified as autonomous (43 or more points on EFAM A exam, applied by the Ministry of Health), and that they have lived in the last 12 months in rural and urban areas of the Bío Bío Region, Chile. Exclusion criteria were set for those who are institutionalized and with diagnosis of mental health disorders that affected their judgment of reality (e.g. schizophrenia, etc.).

399 older adults were chosen, using a non-probabilistic quota sample. We defined proportional quotas to represent each of the 33 municipalities of the Bío Bío Region. It resulted in a sample with a mean age of 72.28, where the majority were women (54.6%) and married (59.1%) (Table 1).

**Table 1:** Characterization of the sample of 399 autonomous Chilean older adults

Variable	Description
Sex	Men: 180 (45.1%)
	Women: 218 (54.6%)
	Not informed: 1 (0.3%)
Age	M = 72.28; SD = 7.01; Min: 60; Max: 99
Marital Status	Single: 36 (9.0%)
	Married: 236 (59.1%)
	Live-in Partner: 9 (2.3%)
	Separated: 30 (7.5%)
	Widowed: 85 (21.3%)
	Not informed: 3 (0.8%)
Educational attainment	With studies: 29 (7.3%)
	Incomplete primary education: 78 (19.5%)
	Complete primary education: 30 (7.5%)
	Incomplete secondary education: 49 (12.3%)
	Complete secondary education: 91 (22.8%)
	Incomplete higher education: 7 (1.8%)
	Complete higher education: 114 (28.6%)
	Not informed: 1 (0.3%)
Perceived state of health	Very poor: 2 (0.5%)
	Poor: 24 (6.0%)
	Regular: 145 (36.3%)
	Good: 201 (50.4%)
	Very good: 25 (6.3%)
	Not informed: 2 (0.5%)
Legally retired	No: 60 (15.0%)
	Yes: 335 (84.0%)
	Not informed: 4 (1.0%)
Remunerated work	No: 320 (80.2%)
	Yes: 71 (17.8%)
	Not informed: 8 (2.0%)

### Instruments

Participants answered the Multidimensional Scale of Perceived Social Support (MSPSS), developed by Zimet, Dahlem, Zimet & Farley [17] and translated into Spanish by Arechabala & Miranda [3]. MSPSS is free to use and does not require a license. This scale presents 12 items that gather information regarding social support perceived by people in three areas: family, friends, and significant others [17]. We used Arechabala’s adaptations where participants must evaluate each collecting agent and answer using a scale following the level which this represents them, ranging from: 1 = Almost never to 4 = Almost always.

A group of experts in the areas of psychology and geriatrics validated the translation into Spanish made by Arechabala & Miranda. Also, this team uses a pilot application in older adults to make the answer options understandable for local population [3].

In addition, participants completed a sociodemographic questionnaire to gather descriptive data of the sample.

Supplementary file 1 shows the Spanish version of these questionnaires applied in this study. Supplementary file 2 shows a proposed English version of them.

### Procedure

First, we obtained the approval from the University of Bio-Bio’s Ethics Committee.

Afterward, a team of trained survey-takers approached older adults following the quotas established per municipality and, within these, randomly chose blocks to contact in their homes the participants who met the eligibility criteria. Once contacted, survey-takers carried out an informed consent procedure, where they explained to older adults the goals of the study, the type of participation requested and the guarantees of voluntariness, confidentiality and anonymity of the provided information. After this, the survey-taker applied the survey orally, registering the answers given.

We performed the survey in January 2019, and the entire application process of the battery of questionnaires took between 50 and 60 min for each participant.

### Data analysis

In order to analyze the factorial structure of the MSPSS as evidence of its construct validity [36], we carried out a confirmatory factor analysis (CFA). Previously, we checked the assumption of multivariate normality through the Mardia test. Due to the 4-points scale used in this version, we used a Weighted Least Squares Means and Variances adjusted estimation (WLSMV), a robust estimation method that can provide accurate estimates and standard errors with ordinal data [37].

We used CFA to compare the goodness of fit of four models for MSPSS supported by previous research:

A) the original 3-factors model (SO, FA, and FR) supported by most of the studies [17, 18, 20, 21, 23–27, 29–31];
B) the 2-factors model where SO merges with FA (SO+FA and FR) that has been found in older adults and people with dependence issues [3, 32];
C) the 2-factors models where SO merges with FR that has been found in teenagers [34], and.
D) the one-factor model that has been found in Hurdu translation [22].
E) We assessed the fit quality of these models by using the following statistics: a) CFI, b) TLI, c) RMSEA and a 90% confidence interval, and SRMR. For CFI and TLI, values of 0.90 or higher indicate acceptable fit, and values of 0.95 or higher indicated good fit. Values of less than 0.08 for RMSEA and SRMR are acceptable and under 0.06 represents excellent fit [38–41].

Finally, we evaluated the reliability of scales using Cronbach’s α and McDonald’s ω reliability coefficients, as the latter offers a less biased estimation of reliability [42].

We used Mplus 8.4 for the data analysis.

## Results

Mardia test showed that coefficients of asymmetry and kurtosis resulted to be non-significant (*p* > .05).

Table 2 shows descriptive statistics of each one of the proposed factors, and evidence that both factors present a negative asymmetric distribution, which is less pronounced in the factor of social support received from Friends. Furthermore, the distribution of the three factors is clearly leptokurtic.

**Table 2:** Descriptive statistics of the factors of the Multidimensional Scale of Perceived Social Support in the Chilean older adults

Factor	Mean	SD	Min	Max	Skewness	Kurtosis	P_25_	P_50_	P7_5_
Family	14.16	2.90	4	16	−1.79	5.46	13	16	16
Friends	12.52	4.05	3	16	−0.93	2.56	10	14	16
Significant others	14.03	3.05	4	16	−1.72	5.24	13	16	16

Table 3 shows the fit indexes of the four evaluated factor solutions. The four models presented CFI and TLI index over 0.95, which showed an excellent fit, but the 3-factors model achieved the better fit of all of them. Also, the SRMR indicated excellent fit only for the 3-factors model (A) and for the 2-factors model that merges Family and Significant others (C). Nevertheless, RMSEA was over the cutoff point in the four cases, showing a poor fit, even when the 3-factors model was closer to acceptable performance.

**Table 3:** A comparison of the fit indexes of 3-factors, 2-factor, and one-factor models for Chilean older adults

Factor	CFI	TLI	RMSEA (90% CI)	SRMR
A. 3 factors	0.991	0.989	0.082 (0.070–0.095)	0.035
B. 2 factors (FR + SO)	0.968	0.961	0.152 (0.141–0.164)	0.087
C. 2 factors (FA + SO)	0.989	0.986	0.091 (0.079–0.103)	0.042
D. 1 factor	0.965	0.957	0.158 (0.147–0.170)	0.100
E. 3 factors^a^	0.991	0.989	0.082 (0.069–0.094)	0.034

Notes: *TLI* Tucker-Lewis Index; *CFI* comparative fit index; *RMSEA* root mean square error of approximation; *CI* confidence interval; *SRMR* standardized root-mean-square residual; *FR* Friend; *SO* Significant other; *FA* Family; ^a^ Correlated errors between items 1–10

After that, we explore the modification indexes, and this analysis suggested that the more important was a correlated error between items 1 and 10 in the factor SO. However, its inclusion in the model only slightly improved the indicators. In fact, RMSEA showed perceptible changes only in the confidence interval.

Due to the model B implies the risk of an overspecified model that could not be replicated in other samples, and it did not represent a significant improve in our indexes, we decided to support the model A.

**Fig. 1:**
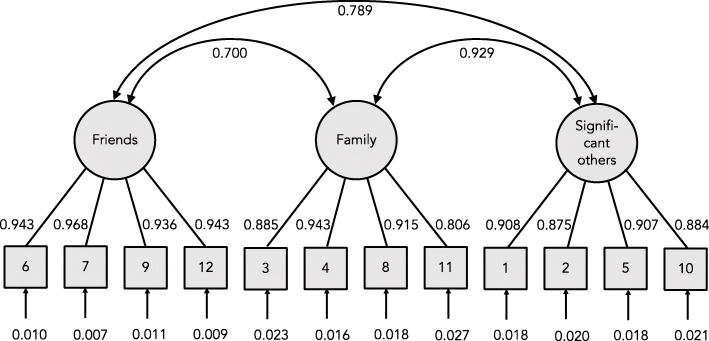
Confirmatory factor analysis of the Multidimension Scale of Perceived Social Support in Chilean older adults

Figure 1 shows the estimated parameters for model A. The standardized loadings were from 0.943 to 0.968 for the factor Friends, from 0.806 to 0.843 for the factor Family, and from 0.975 to 0.908 for the factor Significant others. The correlations between factors were between 0.700 and 0.929. The bottom of the image shows the standardized errors for each item.

Finally, we calculated the reliability for the three factors. For the factor of social support received from the Family, the Cronbach’s alpha was α = 0.858, while the McDonald’s omega was ω = 0.972. For the factor of Friends support, the Cronbach’s alpha was α = 0.941, and the McDonald’s omega was ω = 0.937. And for the factor of Significant other’s support, the Cronbach’s alpha was α = 0.873, while the McDonald’s omega was ω = 0.941.

All of them were qualifiable as good (> 0.8) or excellent (> 0.9) according to the classification of George & Mallery [43].

## Discussion

The validity of an instrument depends on the extent to which it has a theory basis and empirical evidence for the interpretation that is given in one use, in particular [36]. In this case, the possibility of obtaining measurements from the Multidimensional Scale of Perceived Social Support (MSPSS) depends on the extent to which the scientific community has evidence that underpins the existence of these measurements.

The factorial analysis in this study assessed whether MSPSS items expressed latent variables and whether these were theoretically plausible. The results showed that the best organization for the items is three factors, which showed a better fit than the other competing models. However, we cannot overlook that this solution did not achieve an acceptable fit in one of the three employed criteria: RMSEA.

The supported solution differentiates the perceived support from Family, Friends, and Significant others. It is entirely consistent with the original Zimet’s solution from 1988 [17], found in university students from the United States, and subsequently confirmed by studies in different populations from the same country [20, 32], and France [23], Thailand [33], Nigeria [25], España [24], and Colombia [18]. Even previous studies in Chilean older adults that used the same MSPSS adaptation we used in this research have endorsed those 3-factors [19, 31].

In this way, this study leads to confirm the previous evidence that de MSPSS measures are a valid approximation to assess perceived social support, differentiating the three abovementioned sources. Nevertheless, we need new research to evaluate if the frequency 4-points scale proposed by Arechabala [3] is restricting the variability of responses and producing the ceiling effect that Zimet [17] adverted in 1988, but also if Chilean older adults as others in developing countries could have an adequate performance with five or seven alternatives. Arechabala changed the quantity and the type of questions for those Chilean older adults, because they were easier to understand for them. It could be related to the fact that Chilean older than 60 years presents cognitive impairment three times more frequently than the younger population, and its prevalence is thirty times higher in adults over 80 years of age. Moreover, its risk is even higher for retired people [44] that was over de 80% of our sample.

Another problem in our solution was the high correlations between the three-factor (0.700 to 0.929), that were superiors than those informed in previous studies that endorsed the 3-factors model [17, 20, 24, 30, 35], but similar to the correlations reported in Nigerian teenagers, were they were from 0.80 to 0.95 [25]. The highest correlation was between Family’s and Significant others’ factors, putting in doubt it those factors are distinguishable, or it is best a solution the 2-factors solution (Model C), as has been found in the first study with this MSPSS adaptation [3]. As we said, Model C has been found in other studies [3, 32], and it is plausible for older adults given that their progressive functional limitations favor family support [7] and make their contact with friends difficult. However, upon remaining within the close circle of older adults, significant others and family would not require that the elderly move to them. Also, increasing dependence, such as the related to aging, can impact social resources and make less distinguishable the family and significant others [32].

Nevertheless, even if Model C showed a good fit in almost every index and only was under the criterion in RMSEA, like the 3-factors model, the latter showed slightly better performance.

The high correlation between Significant others and Family could be addressed by the solution proposed by Wongpakaran and colleagues in 2012 [19], where they added a note at the beginning of the questionnaire: “Significant other excludes friends and family”, that has shown to improve the comprehension of the “significant other” and has helped to identify the 3-factors solutions in a subsequent study with older adults [33].

This is a way to analyze and improve MSPSS even though it has obtained significant support to endorse a 3-factors solution, with excellent reliabilities, which indicates that the measurements are highly accurate and would present a low error rate associated with the items chosen in the instrument [36]. In fact, in this study0s reliabilities were qualifiable as good or excellent according to George & Mallery guidelines [43].

But another pending reflection is if future versions must consider other possible sources of support like pets, neighbors, and colleagues [12]. This, despite that, the last group is an unlikely source given that older adults tend to be retired. In fact, in this study, more than 80% were retired or had wage-earning work.

Social support reduces mental health issues like depression, stress, anxiety, low self-effectiveness, and social isolation [7, 8, 12], and improves the quality of life of this population [10–12]. So, this evidence endorsing the use of the MSPSS as a 3-factor measurement will support research in the area, to delve deeper into other ways in which social support affects the life of older adults and what the factors predict that the family, friends, and significant others remain close and support this population.

However, it is necessary to consider as limitations of this study that the MSPSS is a measurement of support perceived but not received. Therefore, it has to do with the extent to in which the subject feels that these three groups (Family, Friends, and Significant others) satisfy their needs and not whether these, in fact, do so [10]. But it does not imply perceived social support is less important. On the contrary, perceived social support would have a more significant effect in mental health than objective one [33], it is related to structural and cognitive social capital [9], and empirical evidence reports that higher perceived social support is related to health-related behaviors, less anxiety, lower depression disorders, less utilization of health services, cognitive function, and even lower mortality in older adults [9, 32].

On the other hand, although the Chilean Bío Bío Region is geographically different from the Metropolitan Region where the capital of Chile is located, and where Arechabala & Miranda [3] performed their study, and the cities of Temuco [29] or Chillán [31] were the others study took plase. Compared to Metropolitan Region, Bío Bío is pretty similar at a domestic level: they are two of the three most urbanized and densely populated areas of the country (461.8 inhabitants by km^2^ in the Metropolitan Region and 289.8 inhabitants by km^2^ in the Bío Bío Region). They have very high Human Development Indexes, with 0.874 in the Metropolitan Region and 0.817 in the Bío Bío Region, and have an older adults’ population of slightly over 10 % [1]. For this reason, assessing whether the instrument works similarly in other contexts, both in Chile and in other Spanish-speaking countries, with less urbanization and population density, is still pending.

In the same way, to improve the studies on MSPSS, evaluating its criterion validity with measurements of objective support received and the wellbeing of older adults in the future is pending, as is making a transcultural analysis of the operation of this instrument in different countries and realities for an aging population.

## Conclusions

The results support the existence of three factors for the Multidimensional Scale of Perceived Social Support (MSPSS), differentiating the perceived support from Family, Friends, and Significant others. This solution is theoretically consistent and coherent with the literature, which is why it is presented as evidence in favor of using MSPSS as a 3-factors measurement of the support received. Likewise, its factors present good or excellent reliability, evidencing a high accuracy in their measurements.

## Supplementary Information

**Additional file 1: Supplementary file 1** Spanish version survey.

**Additional file 2: Supplementary file 2** Proposed English version survey

## Data Availability

The datasets used during the current study are available from the corresponding author on reasonable request.

## Abbreviations

MSPSS: Multidimensional scale of perceived social support
TLI: Tucker-lewis index
SRMR: Standardized root mean-square
CFI: Comparative fit index
RMSEA: Root mean square error of approximation
FR: Friend
SO: Significant other
FA: Family
