# Supplementary material for: Multidimensional scale of perceived social support: evidence of validity and reliability in a Chilean adaptation for older adults
Source: BMC Geriatr. 2021 Aug 11;21:461. doi: 10.1186/s12877-021-02404-6 (PMC8359117; doi:10.1186/s12877-021-02404-6)
Supplement: Supplementary file 1 — Additional file 1: Supplementary file 1 Spanish version survey. [file 12877_2021_2404_MOESM1_ESM.docx]

| Cuestionario Sociodemográfico  (Original Espanish Version) | ID: __ __ __ __ |
| --- | --- |

Para finalizar, necesitamos que nos entregue la siguiente información.

| **1. Sexo** | **O** Hombre **O** Mujer |  | **2. Edad** | __ __ años |
| --- | --- | --- | --- | --- |

| **3. Nivel educacional máximo alcanzado** | **O** Sin educación  **O** Educación básica incompleta  **O** Educación básica completa  **O** Educación media incompleta | **O** Educación media completa  **O** Educación superior incompleta  **O** Educación superior completa |
| --- | --- | --- |

| **4. Profesión u oficio** |  |  | **5. ¿Está jubilado/a legalmente?** | **O** No  **O** Sí |
| --- | --- | --- | --- | --- |

| **6. ¿Tiene un trabajo remunerado?** | **O** No  **O** Sí | Si respondió “sí”, ¿cuál? _________________  ¿Por cuántas horas? __ __ horas. |
| --- | --- | --- |

| **7. Estado civil actual** | **O** Soltero (a)  **O** Casado (a)  **O** Conviviente civil  **O** Conviviente, no casado y sin acuerdo legal  **O** Separado (a), divorciado (a)  **O** Viudo (a) |
| --- | --- |

| **8. ¿Cómo percibe su estado de salud?** | **O** Muy bueno  **O** Bueno  **O** Regular  **O** Malo  **O** Muy malo |
| --- | --- |

Recuerde que la información que nos ha entregado es anónima, sólo será utilizada por el equipo investigador y en **ningún momento se hará un análisis individualizado de esta.**

**¡Muchas gracias por su participación!**

**Escala MSPSS**

*Instrucciones:* Utilizando la siguiente escala de 1 a 4, indique su acuerdo con cada afirmación encerrando en un círculo el número apropiado.

|  | CASI NUNCA (1) | **A VECES**  (2) | **CON FRECUENCIA**  (3) | **CASI SIEMPRE**  (4) |
| --- | --- | --- | --- | --- |
| 1. Cuando necesito algo, sé que hay alguien que me puede ayudar | 1 | 2 | 3 | 4 |
| 2. Cuando tengo penas o alegrías, hay alguien que me puede ayudar | 1 | 2 | 3 | 4 |
| 3. Tengo la seguridad que mi familia trata de ayudarme | 1 | 2 | 3 | 4 |
| 4. Mi familia me da la ayuda y apoyo emocional que requiero | 1 | 2 | 3 | 4 |
| 5. Hay personas que me ofrecen consuelo cuando lo necesito. | 1 | 2 | 3 | 4 |
| 6. Tengo la seguridad de que mis amigos tratan de ayudarme. | 1 | 2 | 3 | 4 |
| 7. Puedo contar con mis amigos cuando tengo problemas. | 1 | 2 | 3 | 4 |
| 8. Puedo conversar de mis problemas con mi familia. | 1 | 2 | 3 | 4 |
| 9. Cuando tengo alegrías o penas puedo compartirlas con mis amigos | 1 | 2 | 3 | 4 |
| 10. Hay personas que se interesan por lo que yo siento. | 1 | 2 | 3 | 4 |
| 11. Mi familia me ayuda a tomar decisiones. | 1 | 2 | 3 | 4 |
| 12. Puedo conversar de mis problemas con mis amigos. | 1 | 2 | 3 | 4 |
