# Supplementary material for: Multidimensional scale of perceived social support: evidence of validity and reliability in a Chilean adaptation for older adults
Source: BMC Geriatr. 2021 Aug 11;21:461. doi: 10.1186/s12877-021-02404-6 (PMC8359117; doi:10.1186/s12877-021-02404-6)
Supplement: Supplementary file 2 — Additional file 2: Supplementary file 2 Proposed English version survey [file 12877_2021_2404_MOESM2_ESM.docx]

| SOCIODEMOGRAPHIC QUESTIONNAIRE (Proposed English version) | ID: __ __ __ __ |
| --- | --- |

Finally, we need the following information from you.

| **1. Sex** | **O** Man **O** Woman |  | **2. Age** | __ __ years old |
| --- | --- | --- | --- | --- |

| **3. Nivel educacional máximo alcanzado** | **O** No formal education  **O** Incomplete primary education  **O** Complete primary education  **O** Incomplete secondary education | **O** Complete secondary education  **O** Incomplete higher education  **O** Complete higher education |
| --- | --- | --- |

| **4. Profession or occupation** |  |  | **5. Are yoy legally retired?** | **O** No  **O** Yes |
| --- | --- | --- | --- | --- |

| **6. Do you have a remunerated work?** | **O** No  **O** Yes | If "yes", which one?_______________  How many hours? __ __ hours. |
| --- | --- | --- |

| **7. Marital status** | **O** Single  **O** Married  **O** Live-in Partner  **O** Separated  **O** Widowed |
| --- | --- |

| **8. How do you perceive your health?** | **O** Very good  **O** Good  **O** Regular  **O** Poor  **O** Very poor |
| --- | --- |

Remember that the information you have given us is anonymous, it will only be used by the research team, and we will not make an individualized analysis of this information at any time.

**Thank you very much for your participation!**

**Escala MSPSS**

*Instructions:* Using the following scale from 1 to 4, indicate your agreement with each statement by circling the appropriate number.

|  | ALMOST NEVER (1) | **SOMETIMES**  (2) | **FRECUENTLY**  (3) | **ALMOST ALWAYS**  (4) |
| --- | --- | --- | --- | --- |
| 1. There is a special person who is around when I am in need. | 1 | 2 | 3 | 4 |
| 2. There is a special person with whom I can share my joys and sorrows. | 1 | 2 | 3 | 4 |
| 3. My family really tries to help me. | 1 | 2 | 3 | 4 |
| 4. I get the emotional help and support I need from my family. | 1 | 2 | 3 | 4 |
| 5. I have a special person who is a real source of comfort to me. | 1 | 2 | 3 | 4 |
| 6. My friends really try to help me. | 1 | 2 | 3 | 4 |
| 7. I can count on my friends when things go wrong. | 1 | 2 | 3 | 4 |
| 8. I can talk about my problems with my family. | 1 | 2 | 3 | 4 |
| 9. I have friends with whom I can share my joys and sorrows. | 1 | 2 | 3 | 4 |
| 10. There is a special person in my life who cares about my feelings. | 1 | 2 | 3 | 4 |
| 11. My family is willing to help me make decisions. | 1 | 2 | 3 | 4 |
| 12. I can talk about my problems with my friends. | 1 | 2 | 3 | 4 |
